# IMPLICATIONS OF GLOBAL CLIMATE CHANGE FOR NATURAL RESOURCE DAMAGE ASSESSMENT, RESTORATION, AND REHABILITATION

**DOI:** 10.1002/etc.2036

**Published:** 2012-12-18

**Authors:** Jason R Rohr, Philip Johnson, Christopher W Hickey, Roger C Helm, Alyce Fritz, Sandra Brasfield

**Affiliations:** † Department of Integrative Biology, University of South FloridaTampa, Florida, USA; ‡ U.S. Fish and Wildlife ServiceAnchorage, Alaska, USA; § National Institute of Water and Atmospheric ResearchHamilton, New Zealand; ‖ U.S. Fish and Wildlife Service, Division Environmental QualityArlington, Virginia; # National Oceanic and Atmospheric Administration, Office of Response and RestorationSeattle, Washington, USA; †† U.S. Army Engineer Research and Development Center, Environmental LaboratoryVicksburg, Mississippi, USA

**Keywords:** Contaminant, Baseline, Hazard assessment, Environmental policy, Tipping point

## Abstract

Various international and national regulations hold polluters liable for the cleanup of released hazardous substances and the restoration/rehabilitation of natural resources to preincident baseline conditions, a process often referred to as natural resource damage assessment and restoration (NRDAR). Here, we, the authors, describe how global climate change (GCC) will challenge each of the steps of NRDAR processes and offer eight recommendations to improve these processes in light of GCC. First, we call for a better understanding of the net effects of GCC and contaminants on natural resources. Second, we urge facilities and environmental managers to plan for GCC-related factors that are expected to increase the probability of contaminant releases. Third, we suggest re-evaluating definitions of baseline and reference conditions given that GCC will alter both their trajectories and variability. Fourth, we encourage long-term monitoring to improve the quantification of baseline conditions that will change as climate changes. This will enhance the accuracy of injury assessments, the effectiveness of restoration, and the detection of early warning signs that ecosystems are approaching tipping points. Fifth, in response to or anticipation of GCC, restoration projects may need to be conducted in areas distant from the site of injury or focused on functionally equivalent natural resources; thus, community involvement in NRDAR processes will be increasingly important. Sixth, we promote using NRDAR restoration projects as opportunities to mitigate GCC-related impacts. Seventh, we recommend adaptive management approaches to NRDAR processes and communication of successes and failures widely. Finally, we recommend focusing on managing the stressors that might be exacerbated by GCC, such as pollution and habitat loss, because there is a long history of successfully mitigating these stressors, which can be more easily managed on local scales than climate change. We believe that adoption of these recommendations will lead to a more efficacious NRDAR process, despite the challenges posed by climate change. Environ. Toxicol. Chem. 2013;32:93–101. © 2012 SETAC

## INTRODUCTION

**Fig. 1:**
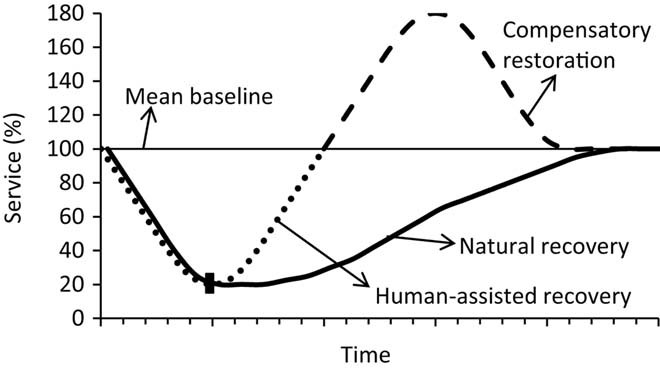
Natural, assisted, and compensatory restoration. Natural restoration or recovery (thick solid line) returns the services of the natural resource to baseline conditions (thin solid line) without the assistance of humans. Human-assisted restoration (dotted line) typically returns the services of the natural resource to baseline conditions sooner than with natural recovery. Compensatory restoration (dashed line) requires the polluter to compensate the public for the time and magnitude of the lost services caused by oil or hazardous substance spill. This often entails improving the services offered by natural resources at ecosystems near the contaminated site.

Various international and national laws and regulations have been enacted to hold polluters liable for the cleanup of released hazardous substances (Table 1), and some further require the polluter to restore any injured natural resources and accompanying services to their baseline condition (the condition but for the hazardous substance release; Fig. 1). For example, the International Convention for the Prevention of Pollution from Ships, known as Marpol 1, provides a basis for holding polluters responsible for the release of hazardous substances into international waters (Table 1). In the United States, the process of holding polluters (responsible parties) liable for the cleanup of certain hazardous substances and the restoration of injured resources and ecosystem services is known as natural resource damage assessment (Table 1) 2–5. The European Union has a similar process that is described in an environmental liability directive (Table 1) 6. In most countries, responsible parties are held liable only for cleanup and sometimes primary restoration, which is restoring the injured resources and services to the baseline condition. In some countries, such as the United States, responsible parties can also be held liable for compensatory restoration 4, 5, 7, which is compensating the public for the interim loss of the injured natural resources and services (Fig. 1).

**Table 1:** Examples of international and national legislation relating to the management of hazardous substances on natural resources

Jurisdiction	Convention/legislation name (abbreviation)	Description	Application	Method of assessment	Reference
International	International Convention for the Prevention of Pollution from Ships	International conventions designed to minimize pollution of the seas, including dumping, oil, and exhaust pollution	Managing marine pollution from ships	Specified lists of chemicals with effects thresholds	1
International	United Nations Environment Programme Liability Guidelines	Minimum guidelines on which national legislation or policies could be based and which would require tailoring to specific national circumstances	Response action and compensation for damage caused by activities dangerous to the environment, taking into account the “polluter pays” principle	Voluntary, meant to serve as a starting point from which national policies could be drafted	55
United States	Comprehensive Environmental Response Compensation and Liability Act (CERCLA), as amended, and Oil Pollution Act (OPA)	Statutory basis for cleaning up hazardous waste sites and oil spills and conducting natural resource–damage assessments	Anywhere hazardous waste or oil is illegally released; establishes liability for injury to, destruction of, loss of, or loss of use of natural resources	Blends science-based assessment with legal and economics-based claims for response, remediation, and restoration/rehabilitation	2, 56
United States	National Oil and Hazardous Substances Contingency Plan, OPA	Legislation covering contaminated “Superfund” sites and natural resource–damage assessment restoration activities	Guides all response and remedial activities	Umbrella authority document for actions under CERCLA and OPA	3, 56
European Union	Directive on Liability to Prevent and Remedy Environmental Damage (European Liability Directive [ELD])	The main objective of ELD is to prevent and remedy “environmental damage”; this is defined as damage to protected species and natural habitats (nature), damage to water, and damage to land (soil)	Parties who carry out certain dangerous activities are strictly liable (without fault) for environmental damage	Requires economic valuation of environmental damage and the different types of remediation and damage to protected species and natural habitats	6
Australia	Environmental Protection and Biodiversity Conservation Act	Overarching Commonwealth Government Act; state governments also have legislation, e.g., New South Wales Protection of the Environment Administration Act	Derivation of guidelines for water-quality management; climate change factored into some natural resource management	Numeric guidelines for water and sediment quality, biological monitoring guidance	57
New Zealand	Hazardous substances and New Organisms Act	Designation of specific hazardous substances and risk assessment related to their use	Setting of environmental limits on selected substances;	Product registration and environmental assessment processes	58
Canada	Canadian Environmental Protection Act (CEPA)	CEPA's purpose is to regulate the behavior of entities in order to promote public safety, protect the environment, and contribute to sustainable development through pollution protection	CEPA also allows the federal and/or provincial governments to sue polluters for the cost of cleanups	Civil liability sections, which are rarely successful; Canada does tend to look to legal precedent rather than legislation	59

Global climate change (GCC) will undoubtedly affect these processes intended to hold polluters liable for cleanup and restoration efforts. The Intergovernmental Panel on Climate Change concluded that human influence on the climate is increasing mean temperatures, the number of areas affected by droughts, the frequency and intensity of heavy precipitation, the likelihood of heat waves, and the occurrence of extreme storms, such as cyclones and hurricanes 8. Indeed, climate change is already causing significant alteration of natural and agricultural landscapes, urban infrastructure, and coastal environments on local, regional, and continental scales 8.

**Fig. 2:**
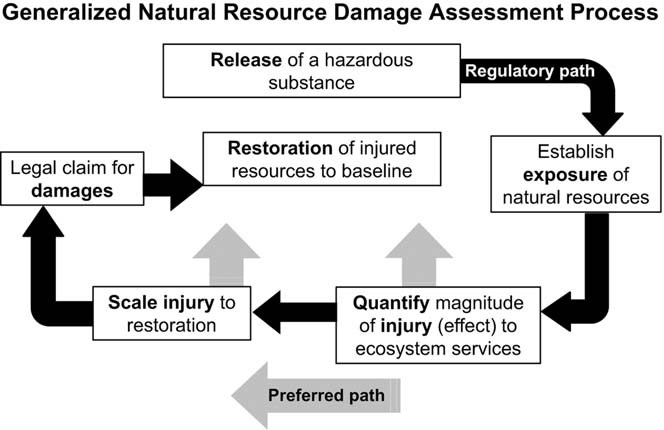
General flowchart of natural resource damage-assessment and restoration/rehabilitation processes around the world. The first step is demonstrating that a hazardous substance was released and reached (pathway and exposure) a publicly owned natural resource (e.g., bird, fish, plant, and public lands). The second step is to establish exposure of natural resources to the hazardous substance. Third is to quantify the magnitude of negative effects on the natural resource (injury). Fourth, decision makers assess a damage claim on the polluter to restore the injured natural resource to baseline conditions. Finally, restoration or rehabilitation is performed. Gray arrows indicate the preferred paths for this process, which focus on beginning restoration actions as early as possible following an accurate understanding of the nature and magnitude of the injury caused by the hazardous substance.

Here, we emphasize how GCC will affect natural resource damage assessment and restoration (NRDAR), which is the process of determining the degree of injury to natural resources caused by a pollutant, the amount of restoration required to return the injured resource to a preinjury condition and the scope of associated environmental rehabilitation efforts. This NRDAR process can be broken down into five general steps: (1) release of a hazardous substance, (2) exposure and injury of natural resources, (3) assessing the extent of any injury, (4) determining the amount or scale of actions required to recover any injured resources and services, and (5) restoring or rehabilitating these resources and services (Fig. 2). Below, we discuss how GCC will influence each of these five stages of NRDAR, with particular emphasis on restoration and rehabilitation efforts. We conclude that each stage will be affected by GCC, and thus, decision makers involved in NRDAR processes worldwide would be wise to plan for and to consider the impacts of GCC.

## GCC AND THE DISTRIBUTION AND PROBABILITY OF HAZARDOUS SUBSTANCES RELEASES

The first step to an NRDAR process is documenting the release of a regulated hazardous substance and attributing this release to one or more responsible parties. As GCC alters geophysical factors, including increasing the frequency and intensity of extreme weather events, there will be an increase in the likelihood of spills, releases of hazardous substances from storage facilities (Table 2), and mobilization of pollutants from contaminated sites 9. Here, we only briefly highlight how GCC will affect the initial release of contaminants in different regions of the world and refer readers to Gouin et al. 10, in this issue, for further details on the effects of GCC on the fate and transport of contaminants.

**Table 2:** Examples of anticipated changes to the distribution, likelihood, and magnitude of hazardous substance releases and spills associated with global climate change

Nature of threat	Geographic region	Likelihood	Net impact	Potential magnitude of impact	Relation to global climate change	Comments	Reference(s)
Trans-Arctic shipping	Arctic	High	Increase	High—shallow water, sensitive species, significant spill response challenges	Seasonal reductions in Arctic ice cover open new shipping lanes	Northern sea route (NSR) 35–60% shorter than Suez or Panama Canal from northern Europe to Asia	11–13
Decreased shipping outside Arctic	Temperate and tropic areas	High	Decrease	Low—relative to total global vessel traffic	Reduced sea ice opening Arctic sea to shipping	Expect increased Arctic shipping to reduce traffic on existing routes	
Oil and mineral exploration and development	Arctic coastal and offshore	High	Increase	High	Consequence of seasonal sea-ice reductions	Arctic rich in coal and mineral deposits; oil/gas shipments via NSR projected to be 40 MT/year by 2020	11, 12, 60
Oil and mineral exploration and development	Arctic inland	High	Decrease	Locally high	Shorter ice road season limits land-based development	Shorter ice road season may make production difficult or uneconomical	11, 60
Erosion	Arctic watercourses and coastal	Occurring	Increase	Locally high—Threatens existing fuel storage infrastructure, landfills, contaminated sites, drilling mud pits	From increased storm energy, coastal rain, tidal surge, and sea-level rise; reductions in permafrost (soil stability)	Average observed erosion rates at some locations equal or exceed 5 meters/year	11, 13
Permafrost melt	Arctic	Occurring	Increase	Locally high—Releases from waste-disposal sites, sewage lagoons, landfills; increased spill risk	Melting releases contaminants “contained” by ice; threat to cities, ports, and pipeline systems (especially Russia)	Northern pipelines and oil infrastructure may experience frost heave, thaw settlement; in discontinuous permafrost slope stability may be affected	13, 14, 60, 61
Increase in category 4 and 5 hurricane frequency; large-magnitude cyclones	Temperate and tropics	High	Increase	High—Hurricane Katrina: release of millions of gallons of oil; hundreds of hazardous materials releases reported	Warmer ocean resulting in longer storm life and/or greater intensity	Observed increase in proportion of category 4 and 5 hurricanes over 30-year period: modeling suggests warming may increase tropical cyclone destructiveness	15–17
Increased intensity and frequency of heavy precipitation events	Tropics and high latitudes	High	Increase	Locally high—Flooding may threaten infrastructure	Sediment scour/deposition decreases reservoir life span, amplifying flood risks, possibly increasing risk of dam failure	Threatened infrastructure includes pipelines, oil and chemical storage sites, industrial facilities, wastewater-treatment plants, and hazardous waste sites	62–64
Decreased precipitation amount and/or seasonality	Temperate and arid areas	High	Decrease	Locally moderate to high	Reduced flood risk in drought areas	Uncertainty: arid areas may, however, experience flash flooding	
Sea-level rise	Global: low-lying coastal areas	High	Increase	Locally moderate to high	While uncertainty exists, most sea-level modeling scenarios project increases	Sea-level rise may inundate coastal contaminated sites, increasing risk to aquatic resources	62
Increased number and intensity of wildfires	Western United States, northern boreal forests, Australia	Occurring	Increase	Locally moderate	Wildfire incidence and/or intensity projected (or currently observed) to increase in some regions.	Fires could potentially threaten oil and gas infrastructure, contaminated sites, and/or hazardous waste-storage sites, increasing spill risk	18, 65, 66

Given the known spatial heterogeneities in the effects of GCC, predictions for how GCC will influence the probability of contaminant releases will likely vary from region to region. For instance, a seasonal reduction in sea ice in the circumpolar north is expected to increase shipment of goods between Europe and Asia via the Arctic Ocean and provide greater marine access to rich Arctic oil, gas, and mineral reserves 11–13. This increased shipping and development may increase the likelihood of spills, with predictable impacts on local ecosystems (Table 2). Moreover, increased thawing of the permafrost in the Arctic will affect soil stability, which may affect infrastructure, including oil and gas facilities and pipelines 14, and could release contaminants currently contained by frozen soils 13. Alternatively, some GCC-related changes may reduce the likelihood of spills; for example, a shorter ice-road season is expected to restrict development of natural resources at some terrestrial Arctic sites, and increased shipment of goods on Arctic routes should reduce the probability of maritime casualties and spills in temperate zones (Table 2).

Tropical and subtropical regions will likely experience increased storm duration and intensity with more destructive hurricanes and cyclones, increased storm surge, and increased intensity and frequency of heavy precipitation 8, 15, 16. These events are expected to exacerbate inland flooding and mudslides, increasing the likelihood of associated oil spills and releases of hazardous materials. The widespread release of contaminants from the flooding in New Orleans during Hurricanes Katrina and Rita illustrates the risks of inadequately contained hazardous substances during an extreme weather event 17. In temperate regions, GCC is expected to increase the frequency and intensity of wildfires 18, potentially threatening infrastructure including oil and gas facilities, pipelines, and hazardous waste sites (Table 2). Finally, across all regions, increased coastal erosion and rises in sea level may threaten fuel storage facilities, pipelines, landfills, and coastal contaminated sites 9.

While acts of God (effects of unanticipated natural disasters or phenomena of an exceptional character that could not have been prevented by exercising due care or foresight) are exempt from many legal NRDAR processes, as weather extremes increase, expectations that infrastructure should be designed to withstand these extremes may grow. Facilities with inadequate design could potentially become more vulnerable to legal action. Consequently, managers of facilities would be judicious to consider strategies that could reduce the likelihood of GCC-related hazardous substance releases, such as construction of stronger dikes, levees, and dams and proactive removal/remediation of landfills and other contaminated sites in areas likely to be threatened by GCC-induced erosion or rises in sea level.

## GCC AND EXPOSURE TO HAZARDOUS SUBSTANCES

Release of regulated hazardous substances and exposure of natural resources to hazardous substances are considered two distinct processes in damage-assessment cases. This is because hazardous substances can be contained and cleaned up before causing widespread exposure of biota and other natural resources. Furthermore, some contaminants degrade rapidly or might be bioavailable only under certain conditions, minimizing exposure 10, 19.

Global climate change will pose challenges to minimizing the exposure of natural resources to hazardous substances. For example, GCC-induced increases in storm and wave intensity may compromise containment and cleanup of spills by conventional means, such as booms, skimmers, and in situ burning, which are often most effective in relatively calm seas and at low wind velocities 20. The environmental fate, transport, and bioavailability of hazardous substances also may be affected by GCC-influenced factors 10, 13, 19. For example, the speciation and retention of elemental contaminants, such as mercury and arsenic, in sediments can depend on biogeochemical and temperature-mediated biological factors 10. Increased temperatures can result in high primary production and altered stratification of water bodies, resulting in greater concentrations of methylmercury, the more bioavailable and toxic form of mercury 21, 22. Already, GCC has increased the release of contaminants from some acid-sulfate soils, which are strongly affected by the degree of soil wetting 23. Furthermore, the toxicity of some hazardous substances increases with temperature 19. In contrast, higher temperatures and associated increases in microbial activity may, in turn, enhance the metabolic breakdown of some hazardous substances and reduce the potential for exposure to biota 10.

## GCC AND INJURY ASSESSMENT

**Fig. 3:**
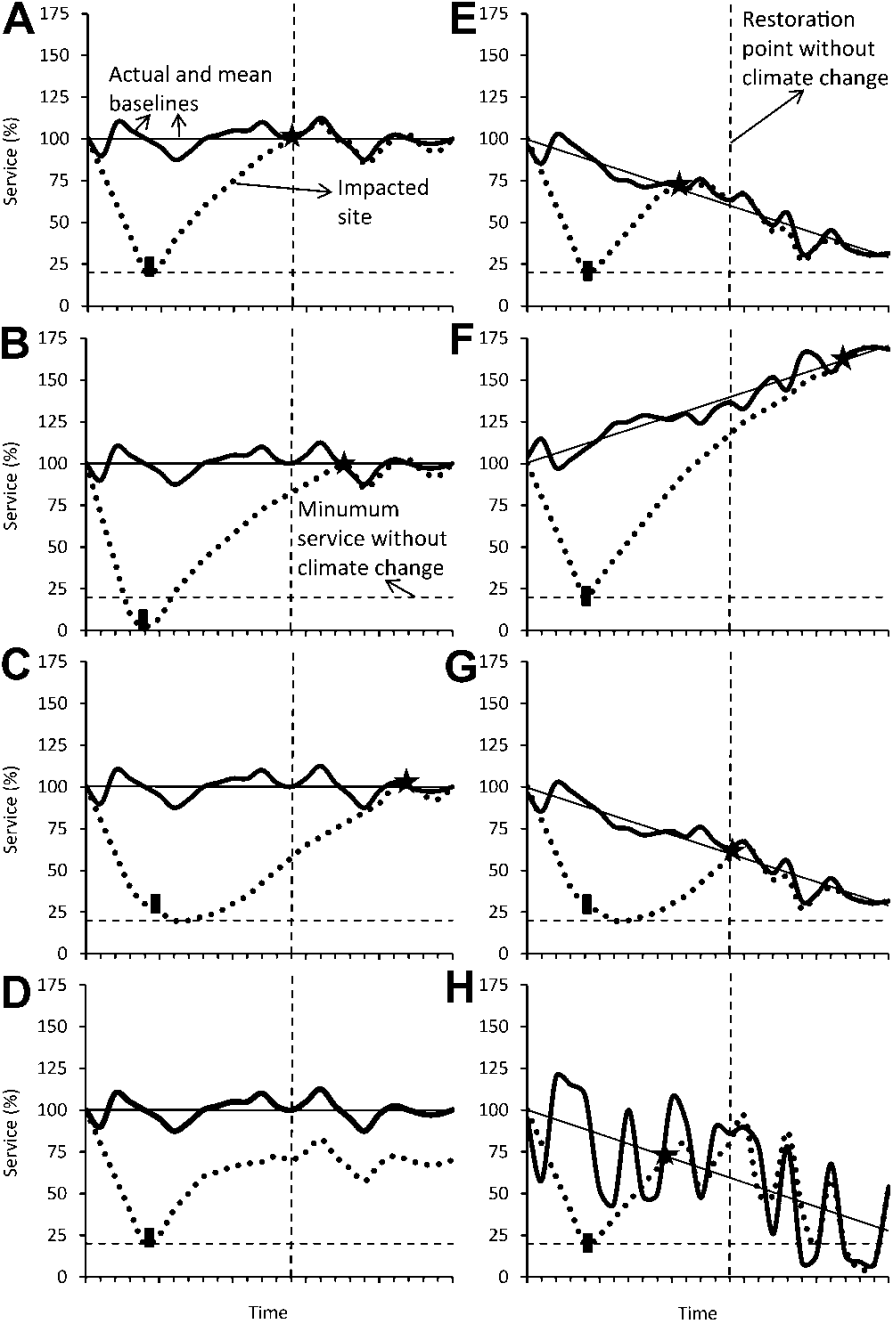
Baseline and primary restoration scenarios without (**A**) and with (**B–H**) global climate change. In each scenario, the hazardous substance release occurs at time zero at the impacted site (dotted line). The rectangle depicts the time at which release of the chemical is ceased and restoration begins (natural or facilitated). The star indicates the point of full recovery/restoration, where the impacted site returns to the baseline condition (mean baseline, thin solid black line; actual baseline, thick solid black line). Thin, dashed, horizontal lines represent the minimum postdamage service in the absence of climate change; thin, dashed, vertical line represents the restoration point in the absence of climate change. Climate change can delay recovery/restoration (star) by additively or synergistically interacting with hazardous substances such that the initial rate of decline of services is greater (**B**) or the rate of recovery is less (**C**) than in the absence of climate change. Climate change might also prevent services from ever returning to preinjury baseline conditions (**D**). Baseline services could also decrease (**E**) or increase (**F**) with climate change, which can accelerate or delay recovery/restoration, respectively. Additionally, there can be combinations of the aforementioned effects that can affect injury assessment and restoration planning, such as climate change–induced decreases in baseline services and rates of recovery/restoration (**G**) or climate change–induced decreases in baseline services and increases in baseline variability that can make it more challenging to assess injury and restoration (**H**). For simplicity, stochastic variability in the contaminated site is not shown until it returns to the baseline condition. This is not meant to be a comprehensive coverage of the potential primary restoration scenarios with and without climate change.

Assessing injury requires documenting an observable adverse effect on the quantity or quality of a natural resource or its associated ecological services relative to a preincident baseline (Fig. 1), with causation linked to a regulated hazardous substance and, under US and EU statutes, attribution of the release to one or more responsible parties 4, 5. Global climate change will affect the assessment of injury by increasing climatic variability and the frequency of extreme climatic events, such as droughts, floods, and hurricanes 8, 24. Many natural resources and the services they provide are influenced by climate; thus, GCC-induced increases in climatic variability and extreme weather events should increase the variability of baseline resources and services (Fig. 3D and H). The more variable the baseline, the more challenging it will be to detect a deviation from baseline caused by a hazardous substance. Hence, GCC-induced variability can increase the threshold of detection during the assessment process, which may result in an underestimate of the magnitude of true injuries. This concern would be most effectively addressed by increasing the frequency and robustness of monitoring efforts to improve the precision of baseline estimates and, thus, increase the probability of detecting deviations from baseline 25.

Furthermore, GCC can make injury assessment more challenging by exacerbating the effects of other stressors on the affected natural resources and services. For instance, evidence is mounting that climate change could increase the frequency of disease in humans and wildlife 26–30 and could cause the decline or extirpation of keystone and foundation species 31 that provide habitat for other species (e.g., overstory trees, corals, and kelp) 32. These multiple stressors can interact additively, antagonistically, or synergistically on the services provided by natural resources 33–36, making it challenging to tease apart the contribution of the hazardous substance to the overall decline in natural resource and services from that of the other GCC-associated stressors 37. Thus, GCC can make it more difficult to definitively and specifically attribute an injury to a release of a hazardous substance 9. Partitioning of variance, however, can provide a tool to differentiate the decline in services that is unique to exposure to the hazardous substance from that which is unique to other stressors or shared by the contaminant and confounding stressors 38, 39.

Reference sites or conditions are often used to determine if a hazardous substance has or is causing an injury to natural resources. The selection of appropriate reference locations will become more difficult if GCC-influenced factors increase background concentrations of contaminants or significantly alter selected reference sites. In addition, GCC-derived weather events that affect contaminant transport and exposure may occur at large spatial scales (e.g., watersheds or regions), affecting downstream receiving areas such as estuaries. These changes may necessitate different spatial and temporal sampling regimes to accurately determine exposure of natural resources to hazardous substances and to assess the magnitude and extent of any injury.

## GCC AND THE SCALING OF INJURY

The process of determining the amount or scale of actions required to recover injured resources and services to baseline and, where required, compensating the public for any lost use of those resources and services is often referred to as scaling injury to restoration. This process may be challenging and time-consuming because it requires the intersection of science, law, and economics 5. Discussing this process in depth is beyond the scope of this article, but one area warranting specific emphasis because of its relevance to the impact of GCC is discounting the future value of natural resources.

Economic studies indicate that the public places a greater value on goods and services available today than on those in the future 40, and accordingly, the costs of injury and the value of restoration projects that occur in the future are often discounted in NRDAR processes 41. Human population pressures and GCC, however, will reduce the availability and quality of many natural resources in the future. These scarcities will lead to higher prices for some natural resources and services, raising both the cost and value of future restoration projects. Consequently, discount rates that are commonly applied to NRDAR processes will need to be reevaluated to account for the fact that GCC is expected to increase the value of future resources.

## GCC AND RESTORATION AND REHABILITATION

Once a contaminant-induced injury has been established and appropriately scaled, a damage-assessment and restoration plan is typically developed and implemented. These plans often include restoration projects that benefit the impacted area and the natural resources and ecological services that were injured. However, when these types of restoration options are not available, restoration projects that are more distant from the injured site and/or are focused on resources that provide similar but not identical services can be considered. Several examples of restoration plans are available from the U.S. National Oceanic and Atmospheric Administration, U.S. Fish and Wildlife Service, and U.S. Department of the Interior Web sites (http://www.darrp.noaa.gov/, http://www.fws.gov/contaminants/Issues/Restoration.cfm, http://www.doi.gov/restoration/index.cfm; also see case study in the Supplemental Data). Importantly, while GCC will profoundly influence restoration efforts and decisions, restoration projects also offer opportunities to mitigate GCC effects.

### Effects of GCC on restoration and rehabilitation

If GCC progresses as predicted in some Intergovernmental Panel on Climate Change scenarios 8, it is likely to have significant implications for restoration actions by potentially increasing the rate of decline of resources and services, increasing the variability in the quality of services, slowing the rate of ecosystem recovery, and increasing the time needed to ensure that long-term recovery has been achieved (Fig. 3). We advocate identifying the specific mechanism(s) by which GCC will affect the restoration process so that decision makers involved in NRDAR processes can implement proactive and adaptive management approaches to improve the odds of restoration success despite the additional stressors presented by GCC.

The toxicity of some hazardous substances increases with temperature 19, 42, and for these specific contaminants, this may result in ecosystem services declining more rapidly under the influence of GCC, thus reaching a lower level by the time restoration commences (Fig. 3B). If this occurs, restoration is likely to be more costly. This scenario, where GCC accelerates the decline of services provided by natural resources (Fig. 3B), might be more problematic than the scenario where GCC affects only the rate of recovery from injury (Fig. 3C). The reason is that, in the former scenario, the minimum level of post-injury services reaches a lower point than in the latter scenario and may result in exceeding an unidentified threshold or tipping point (indicated by horizontal dashed lines on Fig. 3). Exceeding tipping points is not uncommon in ecological systems and can lead to completely new states (Fig. 3D) or functional system collapse 35, 43–45. For instance, if a dispersal-limited species is locally extirpated, it might not recover at all from the hazardous substance exposure, whereas if it only declines, natural recovery might occur quite rapidly if the species has a high reproductive capacity or if there is recruitment from adjacent unaffected habitats 35.

Also, GCC is expected to increase the frequency and intensity of extreme weather events 8, 24, thus increasing the variability of ecosystem services (Fig. 3H). The more temporally variable services are, the more likely that exposure to a hazardous substance combined with stochasticity will exceed a threshold (i.e., tipping point) that either increases restoration needs or prevents successful restoration or rehabilitation (Fig. 3H). Thus, GCC might increase the probability of exceeding ecological thresholds (1) by accelerating the decline of services (Fig. 3B) and (2) by increasing the stochastic variability of services (Fig. 3B).

The key insight from these scenarios is that if GCC generally accelerates the decline in services associated with exposure to hazardous substances, then initiation of cleanup and the restoration process also must be accelerated or the injury (and associated restoration costs), on average, will be greater and successful restoration will be more challenging. Consequently, given these scenarios, it might be prudent for those involved with releases of hazardous materials to expedite cleanup and the damage-assessment process, to begin restoration as soon as possible, and to be watchful for early warning signs that systems are declining rapidly or are approaching tipping points 44–46. If cleanup and restoration cannot be accelerated, injury scaling needs to account for the greater loss in services and, where required, compensatory restoration may need to be expanded to account for these additional injuries.

In the previous scenarios the mean baselines stayed relatively constant through time (Fig. 3B–D), but GCC will almost certainly cause directional changes in baseline variables 43, 47. For instance, many species are shifting their phenologies and moving poleward or to higher elevations as temperatures increase, or they are tracking coastline habitats as sea levels rise 48. Thus, certain baseline services might decrease or increase with time 43, 47, which could reduce or increase restoration efforts, respectively (Fig. 3E and F). This moving target poses considerable challenges for scaling and selecting appropriate restoration projects. For instance, if a site shows evidence of declining services independent of a contaminant release, the contaminant-induced injury might force decision makers to consider replacing the lost resources or services at an alternative site that has, or is anticipated to have, climatic conditions more suited to restoring the injured natural resources 47, 49. With GCC potentially impacting patterns of movement of species and disrupting certain breeding areas or other biologically important habitats (e.g., via rises in sea level or expansions of the range of invasive species), offsite restoration may, by necessity, become more commonplace.

Up to this point, we have independently discussed four mechanisms by which GCC can affect restoration to baseline conditions from a hazardous substance-induced injury: (1) accelerating the rate of decline of services, (2) slowing the rate of recovery of services, (3) increasing variability in services, and (4) altering the mean baseline. It is important to realize that any two or three or all four of these mechanisms can act simultaneously and not necessarily in the same direction, further complicating restoration planning (Fig. 3G and H). Some of these mechanisms might enhance recovery and restoration, whereas others might cause delay (Fig. 3G and H), emphasizing the importance of evaluating the net effect of GCC on these processes 39.

### Using restoration to mitigate the effects of GCC

In making restoration decisions, assessors should consider selecting restoration projects that help to mitigate the effect of GCC. This can be done in several ways, such as by enhancing the sequestration of carbon of an area affected by a hazardous substance release, by adding refugia and/or migration corridors to facilitate organismal movements poleward or to higher elevations 47, by creating or enhancing riparian habitat to mitigate thermal stress in aquatic systems, or by restoring coastal salt marshes, which can reduce erosion of shorelines, attenuate wave action, and limit flooding of coastal communities. Restoration ideally should also strive to create ecosystems that are more resilient to anthropogenic change 47, 50. During restoration processes, ecological resilience can be achieved by incorporating redundancies in species that provide particular ecosystem functions and services so that there are insurances that ecosystems will maintain functions even if some species are lost 51. Similarly, genetic resilience can be accomplished by adding a variety of genotypes within species to restored communities so that there is sufficient genetic diversity available for species to adapt to future natural and anthropogenic changes 52.

### Challenging decisions with socioeconomic ramifications

Future decisions regarding where, what, and how to restore or rehabilitate lost services in the face of GCC will undoubtedly come with increased costs, controversy, and ethical ramifications 49, 50. For instance, given the uncertainties in projections of GCC, it will be challenging to determine how far ecological communities would need to be moved to provide them with a climate to which they are adapted or to predict which assemblage of species and services might eventually prosper at the contaminant-exposed site. Furthermore, the facilitated movement of species can create new pest problems and increase the spread of disease 49. Hence, while these novel restoration approaches have the potential to be better than natural recovery, they also have the potential to be worse.

Restoration actions conducted elsewhere, which focus on different species or that provide different human-use services, may also engender concern because the local community impacted by the hazardous substance may insist that only options that benefit local species and services be considered. Restoration ecologists have stressed that socioeconomic issues warrant as much attention as ecological considerations 53. These socioeconomic considerations will likely grow in the future as GCC complicates restoration efforts.

## CONCLUSIONS AND RECOMMENDATIONS

The effects of GCC on contaminant impacts remain difficult to predict because climate changes can affect the use, uptake, excretion, biotransformation, fate, transport, bioavailability, and toxicity of chemicals 19, 27, 42. Above, we discuss how GCC will likely increase contaminant releases (Table 2), such as by enhancing the frequency and intensity of storm events that may elicit releases from infrastructure and vessels. This enhanced variability in weather is also expected to increase the variability of baseline services provided by many natural resources. Increased variability in baseline services may, in turn, mask the extent of injuries related to hazardous substances (a false negative) and preclude the accurate quantification of resource injuries. In cases where GCC accelerates the decline of services initially caused by the release of a hazardous substance, the polluter may face ever greater damage claims if the pace of assessment and remediation cannot get ahead of the rate of the declining services or if the injured resource is pushed past a tipping point, after which successful primary restoration might not be attainable. As present ecosystems become mismatched with their new climates because of GCC, baseline services are expected to decrease, at least temporarily. In these cases, challenging decisions must be made, such as to shift the restoration of injured natural resources and services to a location with a more suitable climate or to restore the injured resources and services with functional equivalents that are more suitable to the impending climate. Global climate change will increase the uncertainty associated with assessing contaminant impacts and effectively restoring natural resources and services. We conclude that if damage assessment is going to be an effective tool for addressing the natural resource impacts caused by pollution, government agencies implementing NRDAR processes will have to explicitly address and prepare for the effects of GCC.

We offer several recommendations on how to maintain and even improve damage-assessment processes in light of GCC. First, to better inform these processes, we need a better understanding of the net effects of GCC on contaminant-induced injuries to natural resources and ecosystem services 39, 54. Second, we urge facilities and environmental managers to plan for and design infrastructure to withstand GCC-related factors that may increase the probability of contaminant releases, such as enhanced storm intensity and frequency, sea-level rise, coastal erosion, and the melting of permafrost. Third, the definition of baseline and reference conditions will need to be reevaluated given that GCC will alter both the trajectories and variability of baseline ecosystem services. Fourth, we encourage the development of effective long-term monitoring programs to improve the quantification of baseline conditions that will change as climate changes. This will enhance the accuracy of injury assessments, the effectiveness of restoration efforts, and, if possible, detection of early warning signs that systems are approaching tipping points. Fifth, GCC will require consideration of controversial decisions in response to, or in anticipation of, climate change, such as off-site restoration efforts or on-site restoration of functionally equivalent resources; thus, community involvement in the restoration phases of damage-assessment processes will likely become increasingly important. Sixth, GCC will require damage assessors worldwide to think creatively and remember that injuries also can be opportunities to mitigate and prepare for GCC-related impacts. Some of this creativity will be successful, and some will inevitably fail; and many of these successes and failures might simply be due to chance. Hence, our seventh recommendation is to regularly implement adaptive management approaches to more confidently determine which creative solutions actually work and which do not and to communicate these successes and failures widely and publicly. Finally, the task of managing and preparing for GCC is incredibly daunting because it is a planetary problem. This is why our final and most emphatic recommendation is to focus on managing the stressors that could be exacerbated by GCC, such as pollution and habitat loss. We have a long and successful history of mitigating these stressors, which, unlike climate change itself, can be more easily managed on a local scale. Our hope is that damage-assessment regulations and science keep pace with the GCC-related challenges and opportunities we outline in this article and that local efforts to manage pollution and other stressors remain a priority as we attempt to adapt to, and mitigate the effects of, climate change.
