# Supplementary material for: IMPLICATIONS OF GLOBAL CLIMATE CHANGE FOR NATURAL RESOURCE DAMAGE ASSESSMENT, RESTORATION, AND REHABILITATION
Source: Environ Toxicol Chem. 2012 Dec 18;32(1):93–101. doi: 10.1002/etc.2036 (PMC3601426; doi:10.1002/etc.2036)
Supplement: Supplementary file 1 [file etc0032-0093-SD1.doc]

**Lower Duwamish River – Hazardous Waste Damage Assessment Case**

The Lower Duwamish River (LDR) is an approximately seven mile stretch near Elliott Bay, Seattle, Washington, USA that has been contaminated by chronic releases of hazardous substances including: PCBs, PAHs, metals, pesticides, and dioxin/furans. These hazardous substances have been discharged into the river since the early 1900s and government agencies (Trustees) have documented that these substances have injured benthic communities and fish, including the federally-listed threatened Chinook and steelhead salmon. The Trustees, acting on behalf of the public, have worked cooperatively with industries and communities along the LDR to resolve natural resource damage liabilities and to effect restoration of estuarine and riparian habitats. The link <http://yosemite.epa.gov/r10/cleanup.nsf/sites/lduwamish> provides a map of the LDR and identifies Boeing Plant 2, the location of ongoing restoration projects.

The Trustees anticipate that natural and restored habitat in the LDR will be affected by global climate change-driven alterations to sea level, water quality, and water temperature [1, 2]. As part of the natural resource damage assessment (NRDA) process the Trustees developed a Restoration Plan for the LDR that proposes a range of habitat and/or species restoration, rehabilitation, creation, and enhancement projects. This NRDA Restoration Plan is one of the first NRDA Plans to explicitly consider and address the potential impacts from global climate change on proposed restoration projects. The five acre wetland restoration project at Boeing Plant 2 includes elements to account for sea level rise through construction of a range of habitats from shallow subtidal to intertidal to riparian. These suites of habitats were specifically designed to allow for potential upslope migration and to accommodate species specific attributes. In addition, the NRDA settlements obtained for the LDR include 30 years of funding for maintenance, monitoring, and adaptive management actions and additional contributions to a long-term stewardship fund. These provisions for long-term monitoring and management were intentionally designed to address the stresses restored species and habitats will face from climate change.

The National Oceanic and Atmospheric Administration’s Climate Assessment and Proactive Response Initiative (CAPRI) provides a user-friendly geospatial decision-support tool and, through Environmental Response Management Application (ERMA), a web-based support platform that can be used for future restoration planning in LDR and elsewhere in the Puget Sound region (https://www.erma.unh.edu/pugetsound/erma.html). CAPRI, through ERMA, links contaminant sources, climate scenarios, and a vulnerability assessment, enabling interested individuals and communities to visualize and prioritize the potential increased risk to natural resources posed by climate change from the release of hazardous waste and oil, as well as the opportunities for adaptation measures and habitat restoration*.* A more complete description of CAPRI can be found at: <http://www.darrp.noaa.gov/northwest/puget_sound/index.html>.)

[1] King County. 2005. Climate Impacts on Washington’s Hydropower, Water Supply, Forests, Fish, and Agriculture. A report by J.H. Casola, J. E. Kay, A. K. Snover, R. A. Norheim, L. C. Whitely Binder, King County, Seattle, Washington.

[2] University of Washington. 2005. Uncertain future: Climate change and its effects on Puget Sound. A report for the Puget Sound Action Team by the Climate Impacts Group, by A. K. Snover, P. W. Mote, L. Whitely Binder, A. F. Hamlet, and N. J. Mantua, University of Washington, Joint Institute for the Study of the Atmosphere and Oceans, Seattle, Washington.
